# Supplementary material for: Benefits of Participation in Clinical Trials: An Umbrella Review
Source: Int J Environ Res Public Health. 2022 Nov 21;19(22):15368. doi: 10.3390/ijerph192215368 (PMC9691211; doi:10.3390/ijerph192215368)
Supplement: Supplementary file 1 [file ijerph-19-15368-s001.zip › Table S1. Search strings.pdf]

Table S1. Search strings in the umbrella review concerning benefits of participation in clinical trials.

**Database**	**Search string**
Pubmed	(Patient Participation) AND ((Randomized Clinical Trial) OR (Randomized Controlled Trial) OR (randomised trials)) AND ((standard care) OR (non trial) OR (non enrol*) OR (usual practice) OR (routine care) OR (non entry) OR (non participant) OR (outside trial)) AND ((systematic review) OR (meta-analysis)) AND ((Benefits) OR (Patient Harm) OR (side effects) OR (adverse effects) OR (trial effect) OR (protocol effect) OR (ethics)) NOT (protocol [Title]).
Scopus	**(** ALL (("Participation")) AND ALL ((("standard care") OR ("conventional care") OR ("non trial") OR ("non enrol*") OR ("usual practice") OR ("routine care") OR ("non entry") OR ("non participant") OR ("outside trial"))) AND TITLE-ABS-KEY ((("systematic review") OR ("meta-analysis"))) AND ALL ((("Benefits") OR ("Patient Harm") OR ("side effects") OR ("adverse effects") OR ("trial effect") OR ("protocol effect") OR (ethics))) AND ALL (("Randomized Clinical Trial") OR ("Randomized Controlled Trial"))).
Web of Science	Query #8 "standard care" (All Fields) or "non trial" (All Fields) or "non enrol*" (All Fields) or "usual practice" (All Fields) or "routine care" (All Fields) or "non entry" (All Fields) or "non participant" (All Fields) or "outside trial" (All Fields) Query #9 "systematic review" (Title) or "meta-analysis" (Title) Query #10 ALL=("Benefits") OR ALL=(“Patient Harm") OR ALL=("side effects") OR ALL=("adverse effects") OR ALL=("trial effect") OR ALL=("protocol effect") OR ALL=("ethics").
Cochrane Library	"Randomized trial" OR "randomized controlled trial" OR "randomised clinical trial" in Title Abstract Keyword AND "participation" OR "patient participation" OR "benefits" OR "non-particip*" OR "non trial" OR "non enrol*" OR "non entry" OR "outside trial" "patient harm" OR "side effects" OR "adverse effects" OR "trial effect" OR "protocol effect" OR "ethics" "standard care" OR "usual care" OR "usual practice" OR "conventional care" in All Text AND "systematic review" OR "meta-analysis" in Record Title (Word variations have been searched).
