# Supplementary material for: Benefits of Participation in Clinical Trials: An Umbrella Review
Source: Int J Environ Res Public Health. 2022 Nov 21;19(22):15368. doi: 10.3390/ijerph192215368 (PMC9691211; doi:10.3390/ijerph192215368)
Supplement: Supplementary file 1 [file ijerph-19-15368-s001.zip › Table S2. Excluded studies.pdf]

Table S2. Excluded studies and reason for exclusion in the selection process.

Author and year	Title	Reason for exclusion
Joosten et al., 2008	Systematic review of the effects of shared decision-making on patient satisfaction, treatment adherence and health status.	No comparison between participants and non-participants.
Fayter et al., 2007	A systematic review highlights threats to validity in studies of barriers to cancer trial participation.	No comparison between participants and non-participants.
Waters et al., 2012	Weight change in control group participants in behavioural weight loss interventions: A systematic review and meta-regression study.	No comparison between participants and non-participants
Warner et al., 2015	A systematic review of the effectiveness of stroke self-management programs for improving function and participation outcomes: self-management programs for stroke survivors.	No comparison between participants and non-participants.
Booker et al., 2011	A systematic review of the effect of retention methods in population-based cohort studies.	No comparison between participants and non-participants.
Byrd-Bredbenner et al., 2017	Systematic review of control groups in nutrition education intervention research.	No comparison between participants and non-participants.
Koopmans et al., 2012	Non-participation in population-based disease prevention programs in general practice.	No comparison between participants and non-participants.
Phelps et al., 2020	A mixed-methods systematic review of patients' experience of being invited to participate in surgical randomised controlled trials.	Information regarding eligibility criteria and treatment not available for non-participants.
Clarke et al., 2011	Effects on patients of their healthcare practitioner's or institution's participation in clinical trials: A systematic review.	Comparing results of RCTs with and without physician participation. No comparison between participants and non-participants.
Stengel et al., 2006	Are the results of randomized trials influenced by preference effects? Part I. Findings from a systematic review.	No comparison between participants and non-participants
Domecq et al., 2014	Patient engagement in research: a systematic review.	No comparison between participants and non-participants.
Coyle et al., 2020	A systematic review of risk communication in clinical trials: How does it influence decisions to participate and what are the best methods to improve understanding in a trial context?	No comparison between participants and non-participants.

Unger et al., 2019	Systematic Review and Meta-Analysis of the Magnitude of Structural, Clinical, and Physician and Patient Barriers to Cancer Clinical Trial Participation.	No comparison between participants and non-participants.
Mapstone et al., 2007	Strategies to improve recruitment to research studies.	No comparison between participants and non-participants.
Fergusson et al., 2018	The prevalence of patient engagement in published trials: A systematic review.	All patients were trial participants.
Treweek et al., 2013	Methods to improve recruitment to randomised controlled trials: Cochrane systematic review and meta-analysis.	No comparison between participants and non-participants.
Kao et al., 2017	Interventions to improve patient understanding of cancer clinical trial participation: a systematic review.	Not same treatment inside RCT and outside RCT.
Daid et al., 2006	Increasing participation of cancer patients in randomised controlled trials: a systematic review.	No comparison between participants and non-participants.
Myers et al., 2020	Implementation Strategies for Interventions Aiming to Increase Participation in Mail-Out Bowel Cancer Screening Programs: A Realist Review.	No comparison between participants and non-participants.
Lacey et al., 2017	Evidence for strategies that improve recruitment and retention of adults aged 65 years and over in randomised trials and observational studies: a systematic review.	No comparison between participants and non-participants.
Nyman et al., 2012	Older people's participation and engagement with falls prevention interventions in community settings: an augment to the Cochrane systematic review.	No comparison between participants and non-participants.
Tse et al., 2013	Measuring participation after stroke: A review of frequently used tools.	No comparison between participants and non-participants.
Vist et al., 2005	Systematic review to determine whether participation in a trial influences outcome.	Outdated version.
