# Supplementary material for: Benefits of Participation in Clinical Trials: An Umbrella Review
Source: Int J Environ Res Public Health. 2022 Nov 21;19(22):15368. doi: 10.3390/ijerph192215368 (PMC9691211; doi:10.3390/ijerph192215368)
Supplement: Supplementary file 1 [file ijerph-19-15368-s001.zip › Table S3. Results.pdf]

Table S3. Results of the selected reviews and meta-analyses evaluating the benefits of participation in clinical trials.

**Author, year**	**Number of studies (No. participants)**	**Number of studies/comparisons in favor of RCT participation* (RR, SMD, OR; 95% CI)**	**Number of studies/comparisons with no statistically significant differences (NSSD) or in favor of no-participation (RR, SMD, OR; 95% CI) ****	**Meta-analysis**	**Heterogeneity I^2 (%), p-value**
Vist [28], 2008	5 studies with randomized trial participation (412 participants).<br><br>80 studies (86362 RCT participants /57071 non-participants).	None of the studies found significant better outcomes in participants.	NSSD: 5 studies	Not reported	Not applicable
	98 comparisons with a dichotomous outcome.	8/98 comparisons in dichotomous outcomes:<br>RR=0.60; 0.42, 0.86<br>RR= 0.73; 0.56, 0.97<br>RR=0.27; 0.07, 0.99<br>RR=0.81; 0.70, 0.93<br>RR= 0.84; 0.75, 0.95<br>RR= 0.39; 0.18, 0.83<br>RR= 0.59; 0.45,0.78<br>RR= 0.23; 0.07, 0.77	NSSD: 85/98 comparisons in dichotomous outcomes.<br><br>5/98 comparisons found statistically significant better outcomes in non-participants.<br>RR= 2.79; 1.04, 7.53<br>RR= 5.36; 1.66, 7.28<br>RR= 1.51; 1.22, 1.87<br>RR= 1.86; 1.19, 2.92<br>RR= 1.77; 1.12, 2.80	Not reported	Dichotomous outcomes:<br>I^2= 42.2%,<br>$p < 0.00001$
	38 comparisons with a continuous outcome.	3/38 comparisons in continuous outcomes:<br>SMD= -0.37; -0.72, -0.01<br>SMD =-0.85; -1.59, -0.10<br>SMD= -0.80; -1.26, -0.34<br><br>3/37 comparisons found a lower risk of mortality:<br>RR= 0.39; 0.18,0.83<br>RR= 0.59; 0.45,0.78<br>RR= 0.23; 0.07,0.77	NSSD: 30/38 comparisons in continuous outcomes.<br><br>NSSD: 34/37 comparisons analyzing mortality<br><br>5/38 comparisons in continuous outcomes found statistically significant better outcomes in non-participants.<br>SMD= 0.47; 0.14, 0.80<br>SMD= 1.01; 0.05, 1.97<br>SMD= 0.40; 0.28, 0.52<br>SMD= 0.10; 0.04, 0.15<br>SMD= 0.07; 0.02, 0.13	Not reported	Continuous outcomes:<br>I^2= 58.2%,<br>$p < 0.00001$

Fernandes [23], 2014	5 studies with RCT participation (646 participants)<br><br>142 studies (100435 RCT participants /91005 non-participants):	RCTs data not reported	Not reported	Not reported	Not applicable
	21 studies on mortality (53714 participants and 25817 non-participants)	None of the studies analyzing mortality found significant better outcomes in participants	NSSD: 21 studies analyzing mortality.	21 studies on mortality: RR=0.92; 0.78,1.07	Mortality studies: I^2 =84%, $p < 0.001$
	69 studies with dichotomous non-mortality outcomes (30253 participants and 30000 non-participants).	1/69 studies with non-mortality dichotomous outcomes: RR= 0.76; 0.62, 0.92	NSSD: 68/69 studies with dichotomous non-mortality outcomes<br><br>NSSD: 30/48 studies with continuous outcomes.	69 studies with non-mortality dichotomous outcomes: RR=0.99; 0.92,1.08	Studies with non-mortality dichotomous outcomes: I^2=70%; $p < 0.001$
	48 studies with continuous outcomes (20537 participants and 28584 non-participants).	9/48 studies with continuous outcomes:<br>SMD= -0.36; -0.61, -0.12	In 9/48 studies with continuous outcomes found statistically significant better outcomes in non-participants. Studies were stratified by non-participant treatment compared to intervention group:<br>-3 with the same treatment: SMD=0.51; 0.21, 0.82;<br>-4 other treatments: SMD= 0.16; 0.07, 0.25;<br>-2 with treatment non-reported: SMD= 0.35; 0.02, 0.68.	48 studies with continuous outcomes: SMD= 0.04; -0.04, 0.12.	Studies with continuous outcomes: I^2= 88%; $p < 0.001$
Gross [28], 2006	25 studies (18399 participants, 8581 assigned randomly to treatment)	3/25 studies:<br>RR= 0.77; 0.44, 1.34<br>RR= 0.79; 0.32, 1.93<br>RR= 0.52; 0.27,1.01	NSSD: 19/25 studies.<br><br>3/25 studies found statistically significant better outcomes in non-participants:<br>RR= 1.19; 1.03, 1.39<br>RR= 2.07;1.07, 4.02<br>RR= 2.27; 1.06, 4.84	Not reported	Not applicable

Peppercorn [26], 2004	24 articles.	15/23 unadjusted comparisons (No numerical data reported).	NSSD: 5/23 unadjusted comparisons.<br><br>7/23 unadjusted comparisons found statistically significant better outcomes in non-participants. (No numerical data provided)	Not reported	Not applicable
		12/17 adjusted comparisons (No numerical data reported).	NSSD: 5/17 adjusted comparisons.<br><br>5/17 adjusted comparisons found statistically significant better outcomes in non-participants. (No numerical data provided)	Not reported	Not applicable
Braunholtz [27,], 2001	14 studies (27 datasets)	15/27 (56%) datasets.<br>(No numerical data reported)	12/27 (44%) datasets found statistically significant better outcomes in non-participants.<br>(No numerical data reported)	Not reported	Not applicable
Nijjar [24], 2017	21 studies (20160 participants, 4759 outcome events: 1055 and 3704 in RCTs participants and non-participants, respectively).	3/21 studies:<br>OR= 0.15; 0.07-0.30<br>OR= 0.58; 0.41-0.83<br>OR= 0.17; 0.06-0.44	NSSD: 17/21 studies.<br><br>1/21 study found statistically significant better outcomes in non-participants: OR= 2.81; 1.10, 7.16.	OR= 0.75; 0.64–0.87	I^2= 64%

(*) In favor of participation: statistically significant better outcomes in RCTs participants (RR, SMD, OR; 95% CI).
(**) In favor of no-participation: statistically significant better outcomes in non-participants (RR, SMD, OR; 95% CI).
