# Supplementary material for: Benefits of Participation in Clinical Trials: An Umbrella Review
Source: Int J Environ Res Public Health. 2022 Nov 21;19(22):15368. doi: 10.3390/ijerph192215368 (PMC9691211; doi:10.3390/ijerph192215368)
Supplement: Supplementary file 1 [file ijerph-19-15368-s001.zip › Table S4. Overlapping.pdf]

Table S4. Overlapping of the selected reviews and meta-analyses evaluating the benefits of participation in clinical trials.

	Vist, 2008	Nijjar, 2017	Fernandes, 2014	Gross, 2006	Braunholtz, 2001	Peppercorn, 2004
Abraham et al., 2004	1					
Bain et al., 2001	1		1	1		
Karjalainen et al., 1989						1
King et al., 2000	1					
Link et al., 1991	1		1	1		
Akaza et al., 1995			1			
Amar et al., 1997			1			
Andersson et al., 2003			1			
Anonymous, 1971					1	
CASS, 1984	1		1	1	1	
Antman et al., 1985	1		1			
Antman et al., 1983					1	
Ashok et al., 2005	1					
Ashok et al., 2002			1			
Bahamondes et al., 2015		1				
Bakker et al., 2000	1		1			
Balmukhanov et al., 1989	1		1			
Bannister et al., 2001			1			
Baum et al., 1979	1					
Bedi et al., 2000	1		1			
Bell et al., 2000			1			
Berglund et al., 1997	1				1	
Bergmann et al., 1994	1					
Bertelsen et al., 1991					1	
Bhattacharya et al., 1998	1	1	1			
Biasoli et al., 2008			1			

Biederman et al., 1985	1		1			
Bijker et al., 2000	1		1			
Blichert-Toft et al., 1988	1		1	1		
Blumenthal et al., 1997			1			
Boesen et al., 2007			1			
Boezaart et al., 1998	1		1			
Boros et al., 1985						1
Brinkhaus et al., 2008			1			
Burgers et al., 2002						1
Williams et al., 2020				1		
Caplan et al., 1984			1			
Chauhan et al., 1992	1	1	1			
Chesebro et al. 1983			1			
Chilvers et al., 2001	1		1	1		
Clagett et al., 1984	1		1			
Clapp et al., 1989	1		1			
Clemens et al. 1992			1			
Cooper et al., 1997	1	1	1	1		
Cottin et al., 1999						1
Cowchock et al. 1992		1	1			
Creutzig et al., 1993	1		1			
Crowther et al., 2012.		1				
Dahan et al., 1986	1		1			1
Dahlberg et al., 1999						
Dalal et al., 2007			1			
Davis et al., 1985	1				1	1
Decensi et al., 2003			1			
Detre et al., 1999			1			

Dowling et al., 2000						1
Eberhardt et al. 1996			1			
Edsmyr et al.,1978	1		1			
Ekstein et al., 2002	1		1			
Elliott et al., 1996	1					
Emery et al., 2003	1		1			
Euler et al., 2005			1			
Feit et al., 2000	1			1		
Feuer et al., 1994.						1
Forbes et al., 2000	1		1			
Forssell et al., 1989	1					
Franz et al.,1995			1			
Gall et al., 2007			1			
Gesche et al., 2014.		1				
Girón et al., 2010			1			
Goodkin et al., 1987			1			
Gossop et al., 1986			1			
Grant et al.,2008			1			
Greil et al., 1999						1
Gunn et al., 2000			1			
Hallstrom et al., 2003				1		
Helsing et al., 1998	1		1			
Henriksson et al., 1986			1			
Henshaw et al., 1993	1			1		
Heuss et al., 2004	1		1			
Hoh et al., 1998			1			
Howard et al., 2009			1			
Howie et al., 1997		1	1			

Jena et al., 2008			1			
Jensen et al., 2003			1			
Jha et al., 1985					1	
Kane et al., 1988			1			
Karande et al.,1998	1	1	1			
Karjalainen et al., 1989					1	
Kayser et al., 2008			1			
Kazemier et al., 2015.		1				
Kendrick et al., 2001	1		1			
Kerry et al., 2002				1		
Kieler et al.,1998	1	1	1			
King et al., 1997	1			1		
King et al., 2005			1	1		
Kirke et al., 1992			1			
Kirke et al., 1992.		1				
Koch-Henriksen et al., 2006			1			
Lansky et al., 1983	1		1			
Lennox et al., 1979					1	1
Lichtenberg et al., 2008			1			
Lidbrink et al., 1995	1		1			
Link et al., 1986						1
Liu et al., 1998	1					
Liu et al., 2009			1			
Lock et al., 2010			1			
Loeffler et al., 1997			1			
Luby et al., 2002			1			
Macdonald et al., 2007			1			

MACESG, 1992	1					
MacLennan et al., 1985	1	1	1			
MacMillan et al., 1986			1			
Mahon et al., 1996	1		1			
Mahon et al., 1999	1		1			
Marcinczyk et al., 1997	1		1			
Marcus et al., 1997				1		
Martin et al., 1994			1			
Martínez-Amenos et al., 1990	1		1			
Marubini et al., 1996						1
Masood et al., 2002	1		1			
Matilla et al., 2003			1			
Mayers et al., 2001						1
Mayo Group et al., 1992			1			
McCaughey et al., 1998	1		1			
McKay et al.,1995	1		1	1		
McKay et al.,1998						
McKay et al., 1998			1			
Meadows et al., 1983						1
Melchart et al., 2002	1		1			
Moertel et al., 1984	1		1			
Mori et al., 2006	1		1			
Morrison et al., 2002			1			
Mosekilde, et al., 2000	1			1		
Nagel et al., 1998	1	1	1			
Neldam et al., 1986		1	1			
Nicolaides et al.,1994	1	1	1	1		

Ogden et al., 2004	1		1			
Palmon et al., 1996			1			
Panagopoulou et al., 2009			1			
Paradise et al.,1984	1		1			
Paradise et al., 1990	1			1		
Petersen et al., 2007			1			
Playforth et al., 1988	1					
Raistrick et al., 2005	1		1			
Reddihough et al., 1998			1			
Reeves et al., 2004	1					
Reiser et al., 1985					1	
Rigg et al., 2000	1		1			
Rørbye et al., 2005	1	1	1			
Rosen et al., 1987	1		1			
Rovers et al., 2001	1			1		
Roy et al., 2000						1
Salisbury et al., 2002			1			
Schea et al., 1995						1
Schmoor et al., 1996	1			1	1	1
Sesso et al., 2002			1			
Shain et al., 1989			1			
Smith et al.,1990			1			
Smuts et al., 2003			1			
Stecksén-Blicks et al., 2008			1			
Stern et al., 2003		1	1			
Stiller et al., 1989					1	1
Stiller et al., 1994					1	1

Stiller et al., 1999						1
Stith et al., 2004			1			
Stockton et al., 2009			1			
Strandberg et al., 1995	1		1			
Suherman et al., 1999			1			
Sullivan et al., 1982	1		1			
Sundar et al., 2008			1			
Taddio et al., 2006			1			
Tanai et al., 2009			1			
Tanaka et al., 1994			1			
Taplin et al., 1986			1			
Tenenbaum et al., 2002			1			
Toprak et al., 2005			1			
Underwood et al., 2008			1			
Urban et al., 1999	1		1	1		
Van Bergen et al., 1995			1			
Van et al., 2009			1			
Verdonck et al., 1995			1			
Villamaria et al., 1997	1					
Vind et al., 2009			1			
Waard et al., 2002	1					
Wagner et al., 1995						1
Walker et al., 1986	1		1			
Wallage et al., 2003	1	1	1			
Ward et al., 1992					1	1
Watzke et al., 2010			1			
Welt et al., 1981		1	1			
West et al., 2005			1			

Wetzner et al., 1979	1		1			
Wieringa-de Waard et al., 2002		1	1	1		
Wikdahl et al.,1992	1					
Williford et al., 1993	1		1		1	
Winger et al., 1989						1
Witt et al., 2006	1		1			
Woodhouse et al., 1995			1			
World Health Organization, 1988			1			
Wyse et al., 1991			1			
Yamamoto et al., 1992	1		1			
Yamani et al., 2005	1		1			
Yersin et al., 1996	1		1			
Young et al., 1996	1					
TOTAL	85	21	143	25	14	24
N=312; r= 205; c= 6<br>CCA =(N−r) /(rc−r)<br>CCA= 10%						
